# Prevalence of *Babesia bigemina* and *Theileria orientalis* in Domestic Cattle (*Bos taurus*) Across the Three Geo-Climatic Zones of Sri Lanka

**DOI:** 10.3390/ijms27177929

**Published:** 2026-09-05

**Authors:** W. M. Gihani K. Weerasekara, W. M. Chathushka Dhananjaya, W. A. Bhagya Wanasinghe, Viskam Wijewardana, Richard Thiga Kangethe, Thilini A. N. Mahakapuge

**Affiliations:** 1 Department of Veterinary Pathobiology, Faculty of Veterinary Medicine and Animal Science, University of Peradeniya, Kandy 20400, Sri Lanka; gihaniweerasekara96@vet.pdn.ac.lk (W.M.G.K.W.); chathushkadhananjaya7@gmail.com (W.M.C.D.); wabhagyaw@gmail.com (W.A.B.W.); 2 Animal Production and Health Laboratory, Joint FAO/IAEA Centre of Nuclear Techniques in Food and Agriculture, Department of Nuclear Sciences and Applications, International Atomic Energy Agency, 2444 Seibersdorf, Austria; v.wijewardana@iaea.org

**Keywords:** climatic zones, *Babesia bigemina*, *Theileria orientalis*, Sri Lanka, cattle, PCR

## Abstract

*Theileria orientalis* and *Babesia bigemina* are hemoprotozoan parasites that adversely affect domestic cattle (*Bos taurus*) productivity and health worldwide. Rapid climatic changes in different geo-climatic zones observed over the past few years may have altered the distribution and transmission of tick-borne pathogens and vectors. Though these parasites are reported in Sri Lanka in surveillance records, there is a limited understanding of their current distribution pattern. The present study aimed to determine the prevalence and genetic characterization of *B. bigemina* and *T. orientalis* in cattle from three geo-climatic zones of Sri Lanka during the period from January 2025 to March 2026. Out of 384 samples collected from animals (wet, n = 124; dry, n = 208; and intermediate, n = 52 zones) in Sri Lanka, the overall prevalence of 60.2% for *T. orientalis* and 7.6% for *B. bigemina* was revealed using PCR-based detection methods. The highest prevalence of *T. orientalis* was observed in the wet zone (86.3%), while the highest prevalence of *B. bigemina* was 17.3% in the intermediate zone. Co-infections were detected in 5.7% of cattle across all three zones. These results indicate the dominance of *T. orientalis* compared with *B. bigemina* in cattle in Sri Lanka. The observation of co-infections highlights the importance of vector control against vector-borne diseases in Sri Lanka.

## 1. Introduction

Tick-borne hemoprotozoan diseases are strongly affected by environmental and climatic factors, as the survival, reproduction, and transmission of ticks are directly associated with temperature and rainfall patterns [1,2]. Due to the variations in these climatic conditions among different geographical regions, the epidemiology and geographical distribution of tick-borne pathogens have been altered worldwide and documented [3]. In tropical countries such as Sri Lanka, the past few years have been characterized by irregular rainfall, temperature variations, and warming trends that are interconnected with the effects of global warming [4].

Hemoprotozoan parasites, including *Babesia bigemina* and *Theileria orientalis*, are significant tick-borne pathogens that are the causative agents of bovine babesiosis and oriental theileriosis respectively. *T. orientalis* has been reported in cattle, yaks, and buffalo [5], whereas *B. bigemina* primarily infects cattle and water buffalo [6]. Infected animals can develop fever, anemia, and jaundice, and severe cases can result in significant economic losses due to infertility, reduced milk production, weight loss, abortions, and mortality [7,8].

Given the significant effect of these hemoprotozoan parasites on the health and productivity of domestic cattle (*Bos taurus*), it is crucial to have a proper understanding of their pattern of distribution in the three geo-climatic zones: wet, dry, and intermediate zones of Sri Lanka. Based on the Climate Impact Profile (2025) by Geldin Sam [9], the wet zone receives more than 2500 mm of annual rainfall and is characterized by a mean annual temperature of approximately 27–28 °C in the lowland regions. The intermediate zone receives between 1750 and 2500 mm of annual rainfall, with mean annual temperatures ranging from 24 to 28 °C. In contrast, the dry zone receives less than 1750 mm of annual rainfall and experiences relatively higher temperatures, with a mean annual temperature of approximately 28.5 °C in the lowland areas of the Northern Province. According to Abeygunawardena et al., climatic zone variation impacts domestic cattle (*Bos taurus*) farming in the country [10]. Continuation of surveillance data across these three zones is essential for developing and maintaining targeted control measures in the cattle industry.

The prevalence of *T. orientalis* and *B. bigemina* has been shown by epidemiological studies carried out in Sri Lanka, highlighting their widespread occurrence in the populations of both domestic cattle (*Bos taurus*) and water buffalo (*Bubalus bubalis*) in the three geo-climatic zones. Sivakumar et al. [11] reported that 73.1% of the cattle samples collected (n = 316) from Nuwara Eliya (wet zone), Polonnaruwa, Ampara and Jaffna (dry zone) were positive for at least one hemoprotozoan parasite, with *T. orientalis* (53.5%) and *B. bigemina* (30.1%) being the most common across the three districts in Sri Lanka. There was significant geographical variation, with *T. orientalis* being more common in Nuwara Eliya and *B. bigemina* in Polonnaruwa [11]. According to a survey by Sivakumar et al. [12] on water buffaloes (*Bubalus bubalis*) (n = 320) carried out in Polonnaruwa, Mannar, and Mullaitivu (dry zone), the prevalence of *T. orientalis* was high (82.5%), whereas the prevalence of *B. bigemina* (1.6%) was comparatively low. A longitudinal study by Sivakumar et al. [13] during 2014–2015 also demonstrated the endemicity of both *T. orientalis* and *B. bigemina* in the cattle populations (n = 236) in Nuwara Eliya and Polonnaruwa, highlighting the varied prevalence respective to the sampling period. Subsequent investigations reported distinct prevalence of *B. bigemina* in domestic cattle (*Bos taurus*) from Nuwara Eliya, Puttalam (dry zone) and Kurunegala (intermediate zone) [14], and *T. orientalis* in the low country wet zone, Galle [15]. In Sivakumar et al. [16], the presence of *B. bigemina* in different parts of the country was confirmed, with clinical cases reported (75–50%) from Badulla (intermediate zone), Jaffna and Kilinochchi (dry zone). In 2024, a molecular surveillance of cattle (n = 206) from Polonnaruwa and Kurunegala emphasized the prevalence of *T. orientalis* and *B. bigemina* at 47.6% and 6.8% respectively [17]. The overall results emphasized a high prevalence of both *T. orientalis* and *B. bigemina* across the three climatic zones of Sri Lanka, with their abundance varying by geographical region.

Despite several regional studies carried out in the country from 2012 to 2024, there has been no comprehensive molecular study published after 2024 comparing the prevalence and distribution of *T. orientalis* and *B. bigemina* in the domestic cattle (*Bos taurus*) population of the three major geo-climatic zones in Sri Lanka.

Hence, the present study was conducted to examine and compare the prevalence of *B. bigemina* and *T. orientalis* parasite species in cattle in the dry, wet, and intermediate geo-climatic zones of the country and to provide current epidemiological data from January 2025 to March 2026 for the confirmation of the distribution of these hemoparasites in domestic cattle (*Bos taurus*) as a surveillance report.

## 2. Results

**Figure 1:**
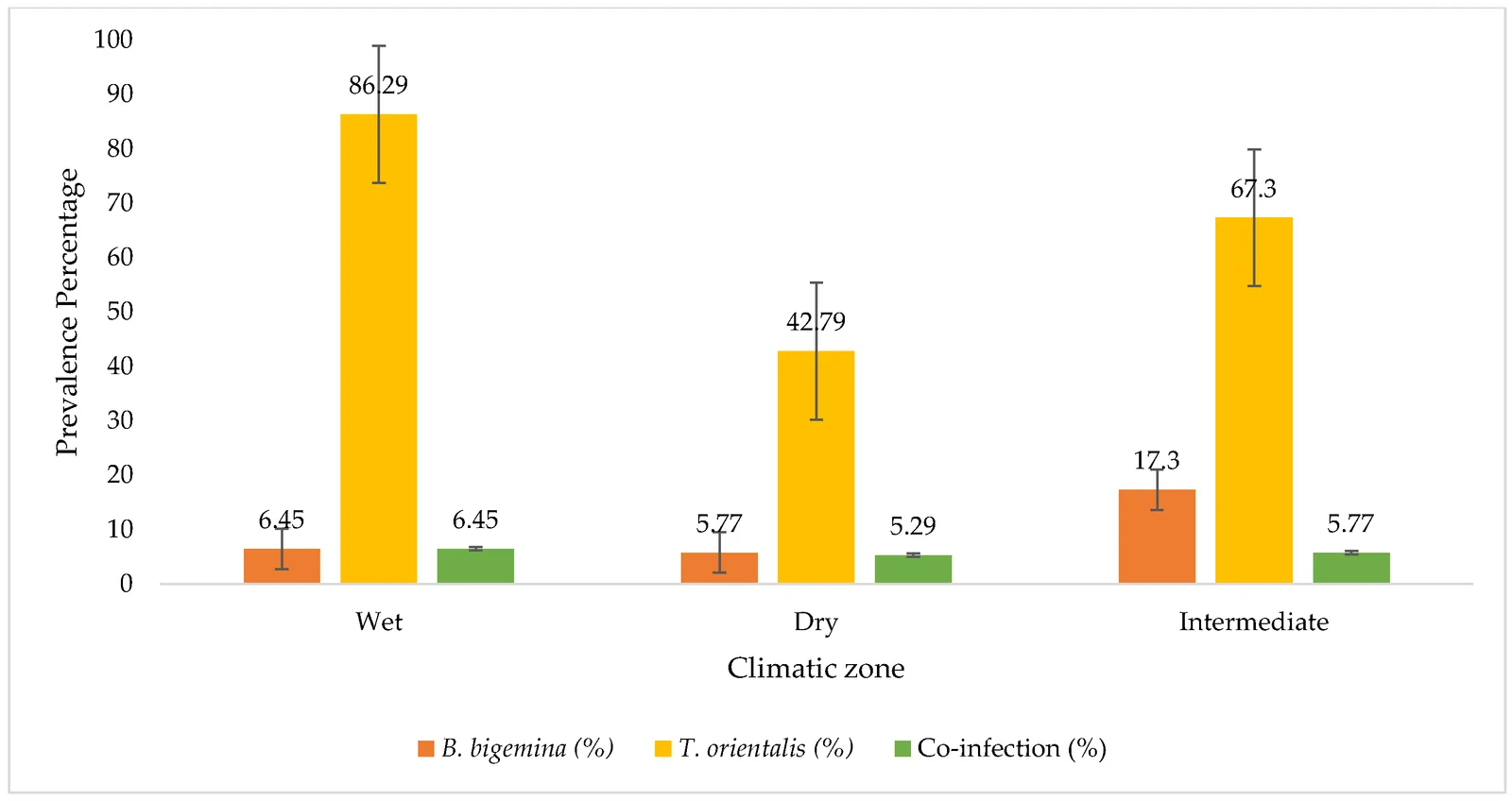
Prevalence of *B. bigemina*, *T. orientalis*, and co-infections in cattle across three climatic zones of Sri Lanka.

The present study detected *B. bigemina* and *T. orientalis* in cattle across three geo-climatic zones in Sri Lanka (Table 1). The most prevalent parasite was *T. orientalis*, detected in 231 animals (60.2%) out of 384 samples. The highest prevalence of *T. orientalis* was observed in the wet zone (86.3%), followed by the intermediate zone (67.3%) and the dry zone (42.8%). In contrast, *B. bigemina* was detected to have a considerably lower prevalence (7.6%). Among the 384 DNA samples analyzed, 22 (5.73%) were co-infected with both parasites, distributed across all three zones: 11 in the dry zone, 8 in the wet zone, and 3 in the intermediate zone, representing 5.3%, 6.5%, and 5.8% of samples per zone, respectively (Figure 1). Co-infection rates highlight overlapping transmission cycles and shared vector exposure across regions.

The prevalence of *B. bigemina* differed significantly among the three climatic zones (χ^2^ = 8.25, df = 2, *p* = 0.016). In contrast, *T. orientalis* showed highly significant variation in prevalence across zones (χ^2^ = 62.62, df = 2, *p* < 0.001), with the wet zone recording the highest prevalence (86.3%).

**Figure 2:**
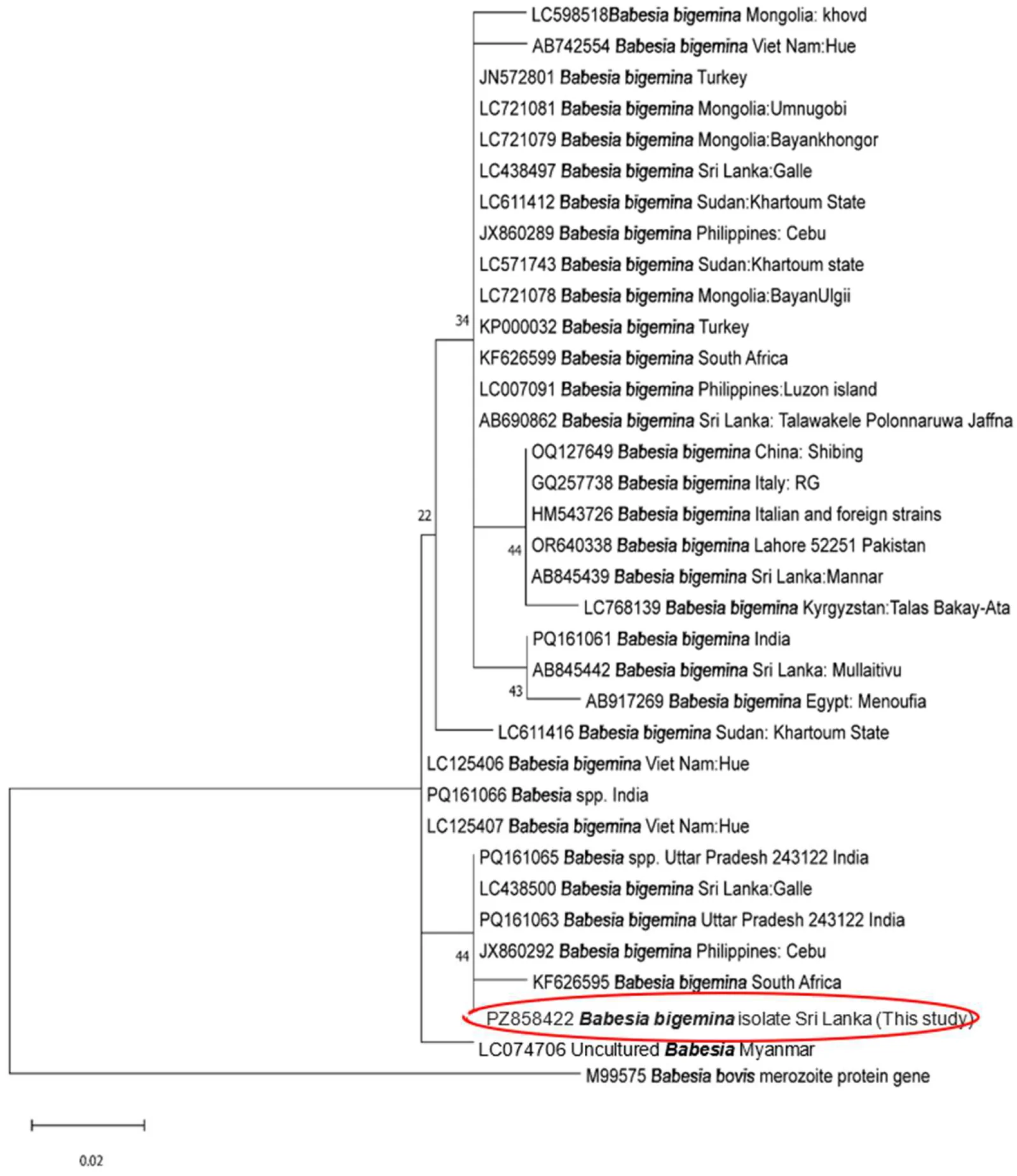
*B. bigemina* AMA-1 gene-based phylogenetic tree. The phylogenetic tree was constructed using *B. bigemina* AMA-1 sequences determined in the present study (labeled “PZ858422 *Babesia bigemina* isolate Sri Lanka” (this study)) and those from several other countries.

The phylogenetic tree constructed for *B. bigemina* (Figure 2) revealed clustering of the Sri Lankan isolate (*Babesia bigemina* isolate Sri Lanka 2025—this study) within the *B. bigemina* clade, clearly separated from the outgroup (*Babesia bovis*; M99575). This confirms the molecular identity of the isolate as *B. bigemina*.

The Sri Lanka 2025 isolate grouped closely with previously reported *B. bigemina* sequences from Uttar Pradesh, India (PQ 161063, PQ 161065), Galle, Sri Lanka (LC 438500), and South Africa (KF 626595). The clustering pattern indicates a close genetic relationship with South Asian strains. Sequences from countries such as Sudan, Egypt, Mongolia, Vietnam, Turkey, and China formed separate sub-clusters within the broader *B. bigemina* lineage.

**Figure 3:**
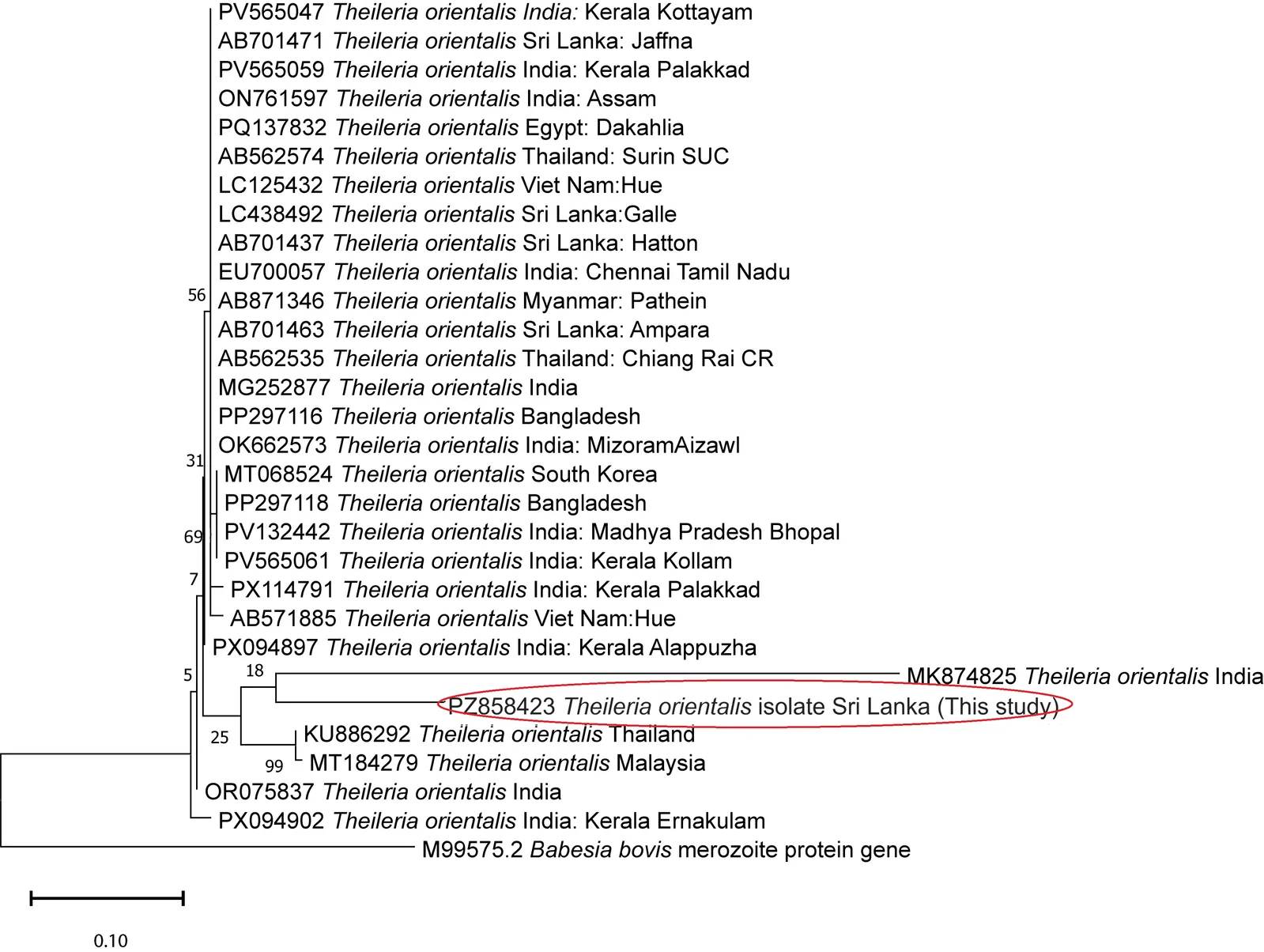
*T. orientalis* MPSP gene-based phylogenetic tree. The phylogenetic tree was constructed using *T. orientalis* MPSP sequences determined in the present study (labeled “PZ858432 *Theileria orientalis* isolate Sri Lanka 2025” (this study)) and those from several other countries.

The phylogenetic tree constructed from *Theileria orientalis* showed that the Sri Lanka 2025 isolate clustered firmly within the *T. orientalis* complex and was clearly distinct from the outgroup (*Babesia bovis*) (Figure 3). The *T. orientalis* Sri Lanka 2025 isolate in this study grouped with MK874825 (*Theileria orientalis* India). It also formed sister clades with KU 886292 (*Theileria orientalis* Thailand) and MT184279 (*Theileria orientalis* Malaysia), indicating regional relatedness.

## 3. Discussion

The present study investigated the prevalence and genetic characterization of *B. bigemina* and *T. orientalis* in cattle across the three major geo-climatic zones of Sri Lanka. Using PCR-based detection and sequencing, we confirmed the presence of both hemoprotozoan parasites in the Sri Lankan cattle population. Our findings not only provide updated prevalence data from 2025 across different ecological settings, but also place the Sri Lankan isolates within their respective phylogenetic clades. This dual approach allows for a clearer understanding of both the epidemiological distribution and the genetic relatedness of the local parasites to previously reported isolates from Sri Lanka and neighboring countries.

Therefore, in the present study, we examined the Sri Lankan domestic cattle (*Bos taurus*) populations from the three climatic zones using previously described PCR assays to understand the current prevalence of *B. bigemina* and *T. orientalis*.

The detection of *B. bigemina* and *T. orientalis* in cattle across various climatic zones in Sri Lanka is correlated with earlier epidemiological studies in the area. A higher level of *T. orientalis* (60.2%) compared to *B. bigemina* (7.6%) can be seen to be mirrored in other locations where *Theileria* spp. predominate over *Babesia* spp. The higher prevalence of *T. orientalis* in the wet zone (86.3%) compared to the intermediate (67.3%) and dry zones (42.8%) is likely because of climatic and environmental factors favoring the vectors causing its spread [15,18].

The comparatively low occurrence of *B. bigemina* can be linked to the current cattle breed, management practices, as well as the diverse vector dynamics across climatic regions. Breeding strategies are zone-specific, with temperate breeds managed intensively in wet zone and indigenous *Bos indicus* (Zebu) breeds managed predominantly in the dry zone [11,19]. Previous studies conducted in Sri Lanka have shown that intensive management in the wet zone is associated with a higher prevalence of babesiosis, and in the dry region, with lower prevalence of babesiosis [11,13]. Additionally, *B. bigemina* infection rates were significantly higher in extensive management systems (32.8%) compared to intensive/semi-intensive systems (5.0%) [15]. The susceptibility of various breeds of cattle to infection with *Babesia* also varies, with *Bos indicus* cattle being more resistant to *B. bigemina* infection than European breeds [20].

The complexity of hemoprotozoan infections in cattle is indicated by the co-infection rate of 5.73%, which is spread across the three zones and may reflect overlapping vectors and transmission cycles. Co-infection, defined in this study as the simultaneous detection of *B. bigemina* and *T. orientalis* in the same cattle, was observed across the studied geo-climatic zones. The detection of both parasites in the same animals highlights the importance of integrated surveillance and vector-control strategies adapted to regional epidemiological contexts [18].

Overall, these results highlight the need for continued monitoring of the prevalence and distribution of a given parasite, which is essential for developing specific disease control programs related to the diverse ecological areas of Sri Lanka.

These findings are also supported by previous molecular epidemiological surveys that show that high rates of *T. orientalis* and lower rates of *Babesia* spp. were detected in apparently healthy cattle populations [11,13,15,16,21]. In addition, a field vaccination program for controlling bovine babesiosis (tick fever) has been specifically designed for temperate breed cattle in Sri Lanka utilizing a live attenuated vaccine of Australian strain *B. bovis* and Sri Lankan strain *B. bigemina*. This vaccination program, in addition to the breed resistance of *Bos indicus* cattle, which are more resistant to infection with *B. bovis* and *B. bigemina* than European breeds, also helps contribute to the reduced prevalence of *B. bigemina* despite its endemic status [16,20,22].

The placement of the local isolate within the *B. bigemina* clade and its close grouping with LC438494 (Galle, Sri Lanka) and PQ161065 (Uttar Pradesh, India) support the local samples’ identification as *B. bigemina* and suggest genetic affinity with nearby South Asian isolates. Such regional clustering of strains has also been documented for other countries in this region, where high transboundary movement of livestock and shared tick vectors may contribute to gene flow between countries [23].

*B. bigemina* has a global distribution, but previous molecular studies have documented low to moderate genetic divergence between the strains of this species compared to other piroplasm species [24]. Molecular studies conducted in Africa and Asia have also documented similar low sequence variability between genetically distant strains of this species [25]. The topology of the present tree also supports previous reports of genetic conservation of this species from different parts of the world.

The phylogenetic position of the local isolate within the *T. orientalis* clade identifies the species and proves a close evolutionary connection with isolates from India (MK874825), Thailand (KU886292) and Malaysia (MT184279). This pattern of clustering indicates that the Sri Lankan isolate is genetically related to Southeast Asian *T. orientalis* populations. The clearer subclade differentiation observed in the present analysis aligns with previous findings demonstrating significant intra-species genetic heterogeneity within the *T. orientalis* complex [12]. The close relationship between Sri Lankan isolates and Southeast Asian strains can be attributed to common ecological conditions, similarities in the species of vectors, or historical cattle movements throughout the area [26,27].

All these findings highlight the dominance of *T. orientalis* over *B. bigemina* in Sri Lanka, the role of ecological and management impact on the prevalence of the parasites, and the complications of co-infections. Further molecular surveillance in different ecological regions is crucial for identifying changing prevalence and distribution patterns and formulating sustainable and region-specific tick-borne hemoprotozoan infection control programs in cattle.

## 4. Materials and Methods

### 4.1. Blood Sample Collection

**Figure 4:**
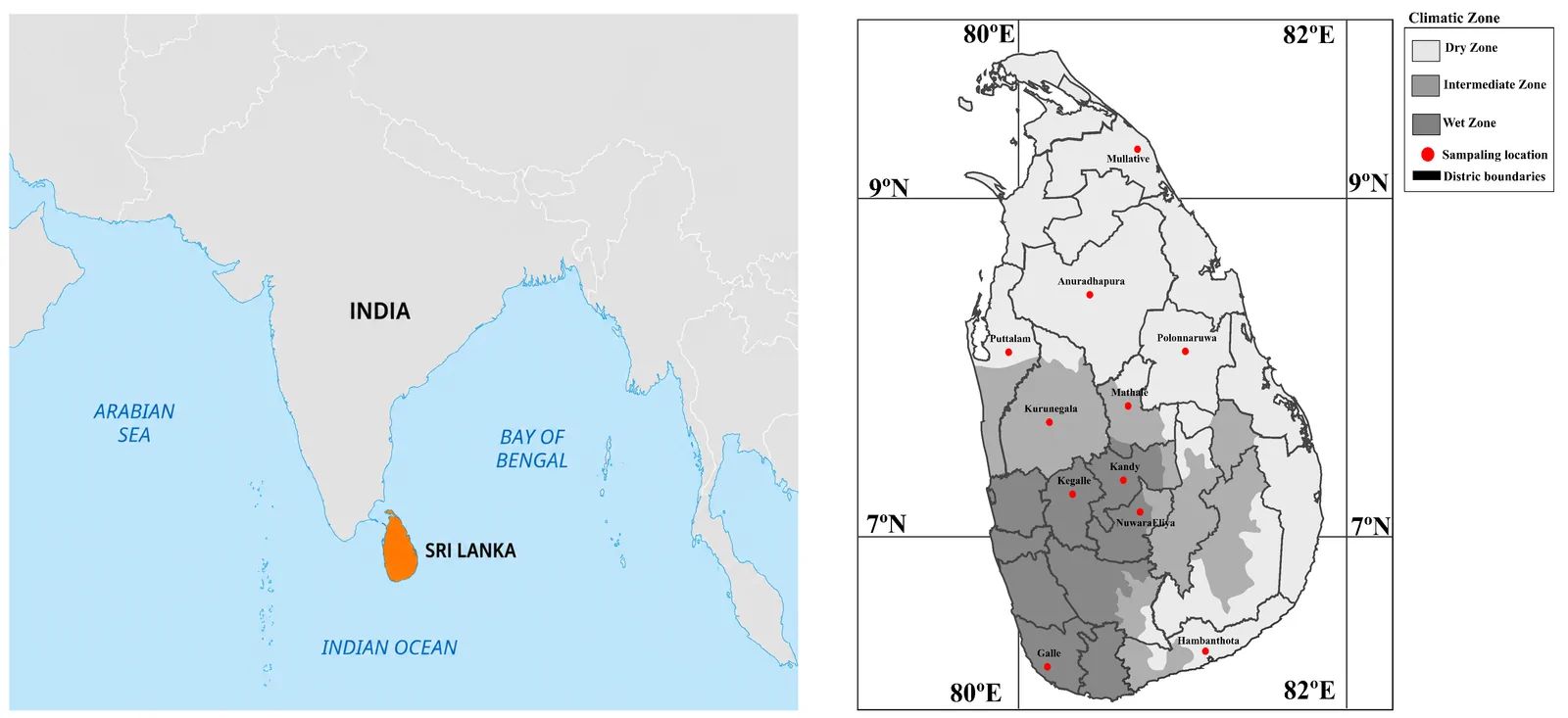
Location of Sri Lanka in South Asia from Britannica and cattle sampling locations within Sri Lanka.

A total of 384 blood samples were collected from cattle in three climatic zones: wet, dry, and intermediate zones in Sri Lanka (Figure 4). All the animals from which the samples had been collected (January 2025 and March 2026) were clinically normal and over one year of age. Animals were selected randomly from each sampling location. Approximately 2 mL samples of whole blood were obtained from the jugular vein of each animal into BD Vacutainer^®^ K2EDTA (3.6 mg) blood collection tubes (Becton, Dickinson and Company, Franklin Lakes, NJ, USA). The blood samples were labeled and stored at −20 °C until DNA extractions were conducted. The total number of samples collected from each climatic zone is summarized in Table 1. Approval for blood sampling was obtained from the Committee on Ethical Clearance on Animal Research, Faculty of Veterinary Medicine and Animal Science, University of Peradeniya, Sri Lanka (Ethical clearance ID: VERC_24_16).

### 4.2. DNA Extraction

DNA was extracted from 200 µL of whole blood from individual cattle using a commercial DNA extraction kit (Biospin Virus Nucleic Acid Extraction Kit, BioFlux, Bioer, Hangzhou, China) according to the manufacturer’s instructions. Briefly, 10 µL of protease K was added to 200 µL of the sample. Then, 200 µL of lysis buffer was added to the sample and incubated for 15 min. Thereafter, 250 µL of ethanol was added, and wash buffers were to carry out three-step centrifugation to discard the flow-through. The DNA samples were eluted with 50 µL of elution buffer provided with the kit. Each extracted DNA sample was labeled and stored at −20 °C until further analyses.

### 4.3. Diagnostic PCR Assays for Identifying Hemoprotozoan Parasites

Previously described parasite-specific PCR assays based on Apical Membrane Antigen-1 (AMA-1) and Major Piroplasm Surface Protein (MPSP) genes were employed to screen all the DNA samples for infections caused by *B. bigemina* [10] and *T. orientalis* [28], respectively (Table 2). Both parasites were screened using single-step PCRs [27,28]. PCR reactions included 12.5 µL of GoTaq Green PCR master mix (Promega, Madison, WI, USA), 1 µL each of forward and reverse primers, 6.5 µL of nuclease-free water (Promega, Madison, WI, USA), and 4 µL of template DNA extracted from cattle blood samples. The PCR conditions for *T. orientalis* identification were carried out for 35 cycles, following an initial denaturation step at 94 °C for 10 min. Each cycle consisted of denaturation at 94 °C for 1 min, annealing at 58 °C for 1 min, and extension at 72 °C for 1 min, followed by a final extension step at 72 °C for 4 min [23].

For *B. bigemina*, the PCR protocol consisted of an initial enzyme activation at 95 °C for 5 min, followed by 35 cycles of denaturation at 95 °C for 30 s, annealing at 68 °C for 1 min, and extension at 72 °C for 1 min. A final elongation step was performed at 72 °C for 10 min [10]. The PCR products were subjected to gel electrophoresis, and the bands were visualized under UV light after ethidium bromide staining. Detection of PCR amplicons close to the expected sizes (i.e., *B. bigemina* 211 bp and *T. orientalis* 776 bp) was considered positive for the respective parasites.

### 4.4. Purification of PCR Products and Sequencing

The QIAquick PCR Purification Kit (Qiagen, Germany) was used to purify the PCR products as per the manufacturer’s instructions. The clean amplicons were then sent to Macrogen Inc. (Seoul, Republic of Korea) to undergo Sanger sequencing, and the sequence obtained was subjected to downstream analysis and confirmation of the target. The nucleotide sequences generated in this study have been deposited in the NCBI GenBank database under accession numbers PZ858422 (*B. bigemina*) and PZ858423 (*T. orientalis*).

### 4.5. Construction of Phylogenetic Trees

The nucleotide sequences generated in the present study were initially analyzed using the Basic Local Alignment Search Tool (BLAST) (http://blast.ncbi.nlm.nih.gov/; accessed on 15 September 2025) provided by the National Center for Biotechnology Information to confirm sequence identity. The *B. bigemina* AMA-1 and *T. orientalis* MPSP gene sequences obtained in the present study, together with reference sequences retrieved from GenBank, were used to construct phylogenetic trees. The maximum likelihood method [29] was applied based on the Tamura–Nei model [30] using MEGA version 11.0 software [31].

### 4.6. Statistical Analysis

The 95% confidence intervals for the prevalence values of each parasite species were calculated using the Clopper–Pearson exact method [32].

The prevalence of *B. bigemina* and *T. orientalis* among climatic zones was compared using the chi-square test (χ^2^) of independence. Differences were considered statistically significant at *p* < 0.05. All statistical analyses were performed using Minitab version 18.0.

## Figures and Tables

**Table 1:** Summary of PCR screening results.

Climatic Zone	Number of Samples	*B. bigemina*		*T. orientalis*	
		Number of Positive Samples	(% ± CI ^a^)	Number of Positive Samples	(% ± CI ^a^)
Wet zone	124	8	6.45 ± (2.83–12.32)	107	86.29 ± (78.96–91.81)
Dry zone	208	12	5.77 ± (3.02–9.86)	89	42.79 ± (35.97–49.82)
Intermediate zone	52	9	17.3 ± (8.23–30.33)	35	67.3 ± (52.89–79.67)
Total	384	29	7.55 ± (5.12–10.67)	231	60.16 ± (55.07–65.09)
*p*-value		0.016		<0.001	

^a^ Confidence interval (*p* > 0.05).

**Table 2:** List of species-specific diagnostic primers used for PCR analyses.

Parasite	Primer Sequence (5′–3′)		Amplicon Size (bp)	Reference
	Forward	Reverse		
*B. bigemina*	TACTGTGACGAGGACGGATC	CCTCAAAAGCAGATTCGAGT	211	[28]
*T. orientalis*	CTTTGCCTAGGATACTTCCT	ACGGCAAGTGGTGAGAACT	776	[27]

## Data Availability

The original contributions presented in this study are included in the article. Further inquiries can be directed to the corresponding author.

## Abbreviations

The following abbreviations are used in this manuscript:

BLAST	Basic Local Alignment Search Tool
PCR	Polymerase Chain Reaction
AMA-1	Apical Membrane Antigen-1
MPSP	Major Piroplasm Surface Protein
